# Ethnic Disparities in Physical, Mental, and Cognitive Health During the COVID-19 Pandemic: a Snapshot of Metro-Detroit

**DOI:** 10.1007/s40615-025-02640-1

**Published:** 2025-10-08

**Authors:** Jasmine M. Cooper, Kristine J. Ajrouch, Simon G. Brauer, Laura B. Zahodne, Toni C. Antonucci

**Affiliations:** 1 Department of Psychology, University of Michigan, Ann Arbor, MI, USA; 2 Institute for Social Research, University of Michigan, Ann Arbor, MI, USA

**Keywords:** Pandemic, Cognitive health, Black, MENA, Depressive symptoms

## Abstract

The COVID-19 pandemic illuminated ethnic and racial disparities in health outcomes within the state of Michigan. These health disparities are evidenced by geographic variability; as roughly half of the COVID cases and more than a third of COVID related deaths in the state occurred in the ethnically diverse Wayne, Oakland, and Macomb counties (New York Times, 2023). However, cognitive health in the context of the pandemic is not well-known, particularly across diverse groups. The current study investigates ethnic differences in health during the COVID-19 pandemic by examining whether there are racial and ethnic differences in physical and mental health status, whether there are ethnic differences in performance in the cognitive domains of episodic memory, working memory, and verbal fluency, and whether ethnicity moderates the association between physical or mental health status and cognitive performance. To examine these associations, we analyzed a sample of 600 Black, White, and Middle Eastern/North African (MENA) older adults from the Detroit Area Wellness Network COVID Supplement, a regionally representative sample of older adults aged 65 and older. Path analyses revealed that Black participants showed a higher burden of chronic illness than other groups, and White participants scored highest in all three domains of cognition. Next, moderation analyses revealed that ethnicity moderated the relationship between mental health status and episodic memory, with a stronger effect in White and MENA participants. These results highlight that the cognitive health of racial and ethnic minority populations of older adults is complex, especially in the context of the pandemic.

## Introduction

In the United States, the COVID-19 pandemic greatly influenced the health outcomes of older adults, as it exacerbated existing chronic illness and depressive symptoms. During the pandemic, those with existing chronic illnesses had less access to regular care from their physicians, which led to the exacerbation of existing illnesses [1] Additionally, the burden of depressive symptoms sharply increased among older adults, especially those who faced social isolation [2]. There is evidence that depressive symptoms and chronic illness predict cognitive performance, as those with higher levels of both show worse cognitive functioning [3]. Although these relationships have been well established in the literature, little is known about the relationship between physical and mental health and cognition in the context of the COVID-19 pandemic, especially in diverse groups.

The current study explores ethnic differences in the association between physical and mental health status and cognitive performance by domain in Black, White, and Middle Eastern/North African (MENA) older adults during the COVID-19 pandemic. Specifically, we investigate whether ethnicity moderates the relationship between chronic illness or depressive symptoms, and three domains of cognition: working memory, episodic memory, and verbal fluency performance.

## Theoretical Frameworks

We integrated two prominent theoretical frameworks to motivate the current study: the Scaffolding Theory of Aging and Cognition-Revised (STAC-r) and the Environmental Affordances Model. The STAC-r framework focuses on the influence of life course factors on brain structure, brain function, and cognition. The Environmental Affordances Model focuses on racial and ethnic differences in physical and mental health outcomes. Although both models address the effects of life course factors on late life development, the STAC-r framework does not include potential influences due to ethnicity, and the Affordances model does not include cognition.

### The STAC-r Framework

The revised Scaffolding Theory of Aging and Cognition (STAC-r) is a model that explains how the cumulative effects of life course factors influence brain function and structure and ultimately cognitive outcomes in older adulthood [4]. Within the larger model, Reuter-Lorenz and Park posit that various life course factors can either positively or negatively influence brain function and structure (neural resource enrichment and neural resource depletion, respectively), which ultimately predict cognitive functioning. Depression and chronic illness are two depleting life course factors within the model that are the focus of the current analyses. Both factors are associated with worse cognitive status in older adults due to their deleterious effects on the brain.

The presence of increased depressive symptoms is associated with worse cognitive performance in older adults. Older adults who exhibit higher levels of depressive symptoms show slower performance and poorer working memory performance than older adults with fewer depressive symptoms [5, 6]. A similar association is reported for episodic memory, as those with higher levels of depressive symptoms show worse performance in both encoding and retrieval [7, 8]. The association between depressive symptoms and verbal fluency seem to be influenced by gender, as the link between increased depressive symptoms and worse cognitive performance is seen primarily in women [9, 10]. The literature suggests that these domain specific deficits in cognitive functioning relate to the broader effect of depressive symptoms on executive functioning, as the effects on the higher level systems of cognition have a top down effect on other systems [11].

Chronic illness is also associated with worse cognitive performance. In particular, people with diabetes and cardiovascular disease are more likely to exhibit worse cognitive outcomes, and those with high blood pressure are likely to show more rapid cognitive decline [12–14]. Similar trends are evident at the domain-specific level, but not all chronic illnesses are associated with each domain of cognition. For example, diabetes has been associated with worse episodic memory performance [15] but is not associated with working memory or verbal fluency performance [16]. Additionally, older adults with hypertension display worse performance on working memory and episodic memory tasks than healthy controls [17, 18], but not for the domain of attention [19]. However, older adults who have had strokes have shown deficits in episodic memory [20], working memory [21], attention [22], and verbal fluency [23].

### The Environmental Affordances Model

The Environmental Affordances Model, developed by Mezuk and colleagues [24], focuses on the interrelation between social contexts, health behaviors, and health outcomes. It is designed to highlight how different racial and ethnic group experiences influence health outcomes as a result of differences in their contextual environment. These differences stem from three facets: 1) ones’ external environment can serve as a source of stress and a source to alleviate stress (affordances); 2) cultural norms and social status influence how people are able to cope with stressors; 3) all organisms work to mitigate stress experienced in their life. Further, the model highlights that ethnic minorities face higher levels of stress due to increased exposure to systemic stressors, which, in turn, leads them to partake in harmful coping mechanisms, including alcohol and drug consumption, and translates to health disparities. These health disparities are then compounded by social, economic, and systematic inequality.

Health disparities manifest as physical and mental health disparities. Regarding mental health disparities, Non-Hispanic White older adults show a lower prevalence of depressive symptoms than their Black, Latinx, and Asian counterparts [25]. Arab American older adult rates of depression are relatively unknown; however, a research on community samples shows that depression ranges from 14 to 50% [26, 27] for depressive symptoms, which is higher than the prevalence for non-Hispanic White (9%), and Black American (16.15%) older adults [25]. However, the way that depression is managed by these groups differs. Despite showing higher levels of depressive symptomatology, Black older adults have been shown to better manage their depressive symptoms [28, 29] than White older adults due to built resilience, as highlighted in the Environmental Affordances Model [24] and increased reliance on social networks. In contrast, Arab Americans face challenges managing their depressive symptoms because they are less likely to be screened for depression and less likely to follow up with a behavioral therapist, although more likely to follow up with a primary care physician [30].

These health disparities also manifest in physical health. Ethnic minority older adults are known to face a higher burden of chronic illness than their White counterparts. Specifically, Black older adults have a higher burden of multimorbidity and develop illnesses earlier in life than their White counterparts [31]. Additionally, diseases such as hypertension and heart disease are more common in Black Americans, when compared to non-Hispanic White Americans [32]. Black and non-White Hispanic older adults are more likely to have diabetes and more likely to have a cardiovascular disorder and diabetes concurrently, when compared to White older adults [33]. Additionally, Black Americans are 30% more likely to have Coronary Heart Disease and 1.3 × more likely to have hypertension [32, 34]. Chronic illness is also prevalent in Arab-American older adults, as the prevalence of diabetes is seen to be higher in Arab Americans living in California and Michigan, when compared to other ethnic groups [35–37].

Differences in health outcomes by ethnicity have important implications for the STAC-r framework, as they highlight factors that may indicate important differences in predictors of cognition. Considering the prevalence of these health conditions may lead to a better understanding of how they differentially affect different groups and how these differences can help to explain variations in cognition.

#### Health Disparities in Michigan

The metro-Detroit area provides a unique context to explore health disparities. The tri-county area, composed of Macomb, Oakland, and Wayne counties, is highly segregated, as suburbanization in the late 1940s influenced White Americans to move from the city of Detroit to suburban areas of Oakland, Wayne, and Macomb counties. Because these federal investments were not afforded to Black residents, most remained in the city of Detroit, contributing to segregation within the metropolitan area [38]. Further, many automotive factories relocated from the city of Detroit to suburban areas, increasing the unemployment rate for Black residents, contributing to the economic decline of the city of Detroit, and exacerbating the existing wealth gap [39]. These effects have had lasting effects on health outcomes of Black residents in the city of Detroit. Those who live in historically redlined neighborhoods report worse self-reported health [40] and show a higher risk of developing and succumbing to cardiovascular disorders [41, 42]. Additionally, those who live in disenfranchised neighborhoods of Detroit report higher levels of depressive symptoms as a result of neighborhood stressors [41, 43]. These effects also extend to cognitive health disparities, as Black residents in metro-Detroit who live in neighborhoods with low socioeconomic status show worse cognitive health [44, 45].

The metro-Detroit area is also unique due to having a large MENA population, a group that is simultaneously growing within the United States and understudied. The region is home to the largest concentration of MENA people in the United States, where residents primarily live in Dearborn, MI, a suburb of Detroit, and in large areas of Macomb and Oakland counties [46]. Historically MENA residents of metro-Detroit, have experienced barriers to access to care, as in 2013 roughly 25% of MENA adults reported not having access to a primary care provider or seeing a physician due to healthcare costs [47]. Additionally, MENA adults report lower rates of cervical and prostate cancer screening, when compared to the entire state of Michigan due to barriers to access to culturally competent care [48]. These barriers to care, along with other risk factors including food insecurity and financial strain were associated with a higher rate of chronic illnesses, including cardiovascular disease and cancer, and depressive symptoms for the MENA population in metro-Detroit [49–51].

#### Health Disparities During the COVID-19 Pandemic

The COVID-19 pandemic provides a unique temporal context due to its illumination and exacerbation of existing health disparities, both within the state of Michigan and nationwide. On a national level, older adults and ethnic minorities showed increased rates of depressive symptoms as a result of pandemic specific factors, which include social isolation due to lockdowns, grief due to loved ones succumbing to COVID-19 and other comorbidities, uncertainty about the pandemic, and limited access to pandemic related information [52, 53]. Additionally, the pandemic increased disparities in cognitive and physical health, as the infections, which predominately affected racial minorities, were associated with increased damage to vital organs and the brain [54]. Further, the pandemic highlighted existing health disparities, as ethnic minorities and migrants within the United States showed higher incidence and severity of COVID-19 as a result of preexisting illness that amplified the effects of infection from the virus [55, 56].

Similarly, the pandemic illuminated health disparities in metro-Detroit. During the pandemic, residents of the city of Detroit were more likely to die from COVID-19, when compared to those in Macomb, Oakland, and Wayne counties, despite having a lower number of cases [57]. Of the COVID-19 related deaths in the city of Detroit, 90% were Black residents. This disparity is driven by residents of Detroit having a higher comorbidity burden, which increased infection related complications, and less access to COVID testing[57]. Additionally, MENA people residing in southeast Michigan were more likely to be infected with COVID-19 when compared to their White counterparts [58, 59]. The pandemic also exacerbated existing health disparities within the region. Ajrouch and colleagues found that Black and MENA older adults reported higher levels of COVID-related stressors, which include infection, income loss, and loss of a loved one, when compared to White residents, and that exposure to these stressors were associated with better cognitive performance only in White participants [60].

## Ethnic Differences in Cognition

Multiple studies have documented differences in cognitive performance across ethnic groups. In particular, Black and non-White Hispanic older adults are roughly 20% more likely to show signs of Mild Cognitive Impairment (MCI) than White older adults [61]. There is also evidence that MENA older adults are more likely to face cognitive difficulties when compared to their White counterparts [62].

Racial and ethnic differences in cognition are seen through neuropsychological testing. When assessing global cognition through the Telephone Interview for Cognitive Status (TICS), White older adults perform significantly better than Black and Hispanic participants [63, 64]. These ethnic differences in cognition are also domain specific, as Black older adults performed worse on episodic memory tests from Weschler’s Logical Memory Story A, The East Boston Memory Test, and the Consortium to Establish a Registry for Alzheimer’s Disease (CERAD), when controlling for age and sex [65]. This trend is also evident in verbal fluency, measured by the animal naming task portion of the CERAD [66], and working memory, measured through the forward and backward digit span [67].

There is limited work that explores differences in performance on neuropsychological testing in MENA older adults, as culturally adapted neuropsychological tests have just recently been made available, including a modified Montreal Cognitive Assessment [68] and comprehensive neuropsychological batteries [69, 70]. However, there is substantial evidence that MENA older adults face worse cognitive health outcomes than their White counterparts. Prior work highlights that MENA older adults show a higher prevalence of cognitive difficulties, including worse memory and difficulties in decision making [71, 72]. These cognitive difficulties are especially prevalent in foreign born MENA older adults [72, 73].

It is important to note that many of the ethnic differences in cognition are at least partially explained by racial and ethnic disparities in education, socioeconomic status, and neighborhood resources [74–76]. This highlights that ethnic and racial differences in performance are largely driven by systemic factors and these factors should be considered as explanatory factors when exploring racial and ethnic differences in cognition.

## Ethnicity as a Moderator of Health and Cognition

When exploring the associations between health status and cognition, it is important to acknowledge that prevalence of depressive symptoms and chronic illness are expressed differently across racial and ethnic groups as a result of environmental and systemic factors, meaning the strength of these relationships may differ across groups. Therefore, it is important to take a nuanced approach when considering how physical and mental health manifests across ethnic groups.

Racial and ethnic inequality affects multiple domains of life, which predict physical and mental health outcomes and ultimately cognition. Socioeconomic status, which encompasses income, education, and social class [77] is one of the most prevalent drivers of inequality across racial and ethnic groups. Black and non-White Hispanic Americans are twice as likely to live in poverty [78], which translates to worse quality education as a result of inadequate funding to schools [79], less access to adequate healthcare and mental health services [80, 81], and higher levels of discrimination, which adversely affect physical and mental health outcomes [82]. These inequalities have the potential to compound physical and mental health problems, thereby strengthening their detrimental impact on cognitive outcomes. Because racial and ethnicity based inequalities have such an influence on health outcomes and their functional consequences, it is important to consider them when exploring the relationship between health and cognition, as the strength of these relationships may be dependent on race.

## Research Questions

We utilize the work of Ward and colleagues [83] as a guiding approach to our research questions. To fully understand disparities in cognitive health, we identify 1) whether there are differences in the exposure (physical and mental health status), 2) whether there are differences in the outcomes (cognitive health), and 3) whether the magnitude of the relationship between physical/mental health status and cognitive health differ by race.

- RQ1: Are there ethnic differences in physical and mental health status?
- RQ2: Are there ethnic differences in cognitive performance?
- RQ3: Does ethnicity moderate the relationship between physical and mental health status and cognitive performance?

Based on previous literature, we hypothesize that those of MENA and Black descent will show a higher prevalence of chronic illness and depressive symptoms due to systemic influences as outlined in the Theoretical Frameworks section. Additionally, we hypothesize that there will be ethnic differences in cognitive performance, with White participants performing better across all domains of cognition, compared to Black and MENA participants. Lastly, we hypothesize that the relationship between health status and cognitive performance will differ by ethnicity, with there being a stronger association for Black and MENA participants in comparison to their White counterparts.

## Methods

### I. Participants

We used data from the Detroit Area Wellness Network–COVID-19 Supplement (DAWN-CS) project. Participants in the DAWN-CS were recruited from an existing longitudinal cohort study [84], supplemented by address-based and respondent-driven sampling in order to obtain a racially/ethnically balanced sample of Black, MENA, and White older adults aged 65 +. With regard to the address-based sample, the study used public, aggregate data from the US Census Bureau to identify high-density areas of Black and MENA adults residing in southeast Michigan. The identified tracts were then matched to the US Post Office Computerized Delivery Sequence file, limited to all addresses within selected areas. Addresses were sampled with known probabilities of selection from the sampling frame to represent the target region. People aged 65 + within screened households were then selected with probability to allow appropriate inference to the target population. With regard to respondent-driven sampling [85], Black and MENA participants who completed the study were given the option to refer other individuals who may be interested in participating. Of the entire sample, 66 participants were recruited through respondent driven sampling, with 34 of these participants being recruited by 26 people in the address-based sample. Informed consent was obtained from all participants. Inclusion criteria for DAWN-CS were age 65 +, fluency in English or Arabic, and identifying as MENA (determined by a combination of self and parents’ ethnic identification), Black or White. The overall respondent response rate was 64%. One person who was eligible to participate because they screened in as MENA was excluded because they did not speak English or Arabic.

The study limited the analytic sample to respondents who reported that they had not been told by a doctor or medical professional that they had Alzheimer’s disease or related dementia. The final sample was *N* = 600 (MENA *n* = 199; Black *n* = 205; White *n* = 196). All participants completed a 30-min telephone survey from December 2020 through October 2021. The period within which data were collected did not include the period of confinement due to the COVID-19 pandemic (which ended in the summer of 2020). Nevertheless, the subsequent months involved changing situations and beliefs about how best to contain the virus. December 2020 is when the COVID-19 vaccine became available for emergency use [86]. This time period also saw the emergence of new variants, face mask mandates, and record-level death tolls, especially in communities of color [86]. The world continued to face challenges arising from the pandemic, needing to adapt to the changing situation.

## Measures

### A. Cognition

Cognition was assessed through three measures; The CERAD word list learning test, the Montreal Cognitive Assessment (MoCA) number span subtest, and Animal Fluency. These tests were administered in the preferred language of the participant (English or Arabic). The Arabic version of these tests, which were constructed by our research group, went through extensive review to ensure they are equivalent in validity and reliability to the English version and followed the methodology outlined in [69]. When selecting neuropsychological tests for this study, we selected items that would be suitable to be administered over the phone. To ensure that the neuropsychological battery was not biased toward one cognitive domain, we chose constructs that correspond to distinct cognitive domains. This process for selection is further outlined in Zahodne et al. [69].

### CERAD (Episodic Memory)

Participants completed four total CERAD trials; three which were done immediately and one that was delayed. For the immediate trials, participants were read a list of ten words. The list of words was read slowly at one word every two seconds. At the end of each immediate trial, participants were instructed to recall as many words as they could remember. Participants were instructed to not write any words down. The immediate trials were completed consecutively.

The delayed trial was completed approximately 3–5 min after the immediate trials. During this delay, participants were asked some demographic questions. Participants were not read the list again but were asked to recall as many words as they could from the list.

To create one composite score, we added and standardized the immediate trials, then standardized the delayed trial. Finally, these standardized scores were averaged and standardized.

### MoCA Number Span (Working Memory)

Participants completed two MoCA number span trials; one trial in which they had to repeat a set of numbers forward and one trial where they had to repeat a different set of numbers backward. The MoCA forward trial included five digits which were read slowly by the interviewer at one digit per second. For the MoCA backward trial, participants were assigned three digits, which were read slowly at one digit per second. The purpose of this task was to measure attention and working memory skills.

Number span performance was scored on a pass/fail basis, meaning that if a participant repeated one digit incorrectly, they failed the entire trial. Both the forward and backward trials were combined in order to create a single working memory score. Participants could receive a 0, which represents failing both trials, a 1, which represents passing one trial and failing the other, or a 2, which represents passing both trials.

### MoCA Category Fluency (Animal Fluency)

Participants were given two trials to assess their verbal fluency, one practice trial and one experimental trial. The practice trial involved participants naming as many clothing items as they could within 20 s.

During the experimental trial, participants were given types of animals as a category and were instructed to name as many as they could in one minute. If responses were not given within 15 s, participants were probed with either “Tell me all the animals you can think of” or “any others?”. The raw number of items generated during the experimental trial served as the verbal fluency score.

### B. Physical Health (Chronic Illness)

To determine whether participants have any existing chronic illnesses, participants were asked whether they had been diagnosed by a physician with any of the following: strokes, heart disease, diabetes, dementia, or high blood pressure. Those who reported a diagnosis of dementia were excluded from analyses. There were five participants who reported a diagnosis. To create a score for physical health status, the number of chronic illnesses, besides dementia, were summed and ranged from 0 to 4.

### C. Mental Health (Depressive Symptoms)

Presence of depressive symptoms was measured through administration of the Center for Epidemiologic Studies Depression Scale 10 (CES-D-10). The CES-D 10 is an abbreviated version of the full scale, which has 20 items. To assess depressive symptomatology, participants had to answer 10 items with ratings “Rarely or none of the time (less than 1 day)”, “Some or a little of the time (1–2 days)”, “Occasionally or a moderate amount of time (3–4 days)” or “Most or all of the time (5–7 days)”. Items include questions such as “I was bothered by things that usually don’t bother me” and “I enjoyed life.” Out of the ten items, there were 2 positive items that reflected happiness, and 8 items that represent negative, unhappy symptoms. The positive symptoms were reverse coded during analyses.

For scoring, participants received zero points for answers of rarely or none of the time, 1 point for answers of some or a little of the time, 2 points for answers of occasionally or a moderate amount of time, and 3 points for answers of most or all of the time. The scoring of positive items is reversed. Possible range of scores is zero to 30, with the higher scores indicating the presence of more symptomatology, *α* = 0.88 [87]. For our main analyses, depressive symptoms were treated as a continuous variable. However, for the purpose of describing the sample demographics, we dichotomized depressive symptoms based on a standard clinical threshold. Participants with scores below 10 were categorized as below the threshold for depression, while those with scores of 10 or higher were categorized as above the threshold.

### Race and Ethnicity

Participants were first asked to self-identify with one or more racial and ethnic categories. They were then asked how their mother and father (separately) identified using the same categories. Finally, they were asked their mother’s and father’s ancestry (separately). Respondents were categorized as Black if they personally identified as such. They were categorized as MENA if they or their parents identified as MENA or their parents had MENA ancestry (22 countries and 6 sub ethnic groups, e.g., Chaldean/Assyrian), and the participant did not identify as Black. Finally, they were categorized as White if they identified as White and were not already categorized as Black or MENA. For our analyses, White participants were coded as 1, Black participants were coded as 2, and MENA participants were coded as 3. The resultant racial/ethnic categories are conceptualized as social rather than biological constructs that represent imperfect proxies for experiential factors that are racially/ethnically patterned.

### D. Covariates

Language, gender, and age served as covariates.

### Language

To code language, participants were scored a 0 if they elected to complete the study in English and a 1 if they elected to complete the study in Arabic.

### Gender

Participants self-reported their gender and were coded as 0 if they were male and coded as 1 if they were female.

### Age

Age was measured through date of birth. Participants provided the month, date, and year they were born. The age was then calculated by subtracting the date of birth from the date of the interview.

### E. Demographic Factors (Not included in main analyses)

Although income and education were not included in our multivariate analyses, as a result of these variables being mediators of the effects of race and health status, we included them as demographic variables to characterize our sample. [88]

To measure income level, participants were asked to report whether their monthly income fell between the ranges of < $500, $501–1000, $1001–2000, $2001–3000, $3001–5000, $5001–10,000, or $10,001 <. These ranges were coded 1–7.

To measure education level, participants were asked how many years of school they completed. Scores ranged from 0 to 17, with each score indicating the number of years of education. Scores ranging from 0 to 12 indicate K-12 education or its equivalent, 13–16 indicate college-level education, and a score of 17 represents post graduate education.

### Analysis Plan

We computed univariate statistics, including means and standard deviations, for the entire sample. To explore whether univariate differences of age, gender, education, and income were significant by ethnic groups, we ran one-way ANOVA models and post-hoc Bonferroni analyses.

To answer our three research questions, weconducted path analyses through the R version 4.4.2 using Lavaan package version 0.6–17, where we performed moderation analyses for each of the domains of cognition. To account for missing cases, we utilized the full information maximum likelihood (FIML) command. FIMLallowed us to use all available data without imputation or listwise deletion, which allowed us to maximize our sample size and reduce bias in our sample. We used FIML rather than listwise deletion to ensure that we did not see a reduction in our sample size and to reduce bias where datawere notmissing at random. When utilizing FIML, we treated all predictors and outcomes as endogenous. We excluded income and education from analyses, as our goal for this paper was to quantify the total racial disparity and by controlling for these factors, we underestimate the racial disparity [89].

To answer RQ1, we ran a set of models that explored whether there were ethnic differences in physical and mental health status, where depressive symptomatology and chronic illness were outcome variables, ethnicity was our main predictor, and age, gender, and language were covariates. To answer RQ2, we ran a set of models that explored whether there were ethnic differences in cognitive performance, where episodic memory, working memory, and verbal fluency were outcome variables, ethnicity was our main predictor, and age, gender, and language were covariates. To answer RQ3, we ran a set of models examining whether ethnicity moderates the relationship between physical and mental health status and cognition. Episodic memory, working memory, and verbal fluency served as outcome variables. Health status and ethnicity were the main predictors, with age, gender, and language included as covariates. To test for moderation, we included multiplicative interaction terms between ethnicity and health status. All variables were centered prior to creating the interaction terms, and dummy-coded variables were created for Black and MENA participants. Additionally, we created contrasts to compare the slopes of Black and MENA participants.

## Results

### Descriptive Statistics (Table 1)

#### Age

The average age of the entire sample was approximately 73 (sd = 6.58) years old. The White sample had the oldest average age of 74 (sd = 7.28), followed by the Black sample with an average age of 72 (sd = 5.95), and the MENA sample with an average age of 71 (sd = 6.07). These age differences were significant across ethnicity, F(2,571) = 11.1, *p* < 0.001, *R*^2^ = 0.03. Post hoc Bonferroni analyses revealed that these differences in age significantly differed between the Black and White groups and the White and MENA groups. There was no significant difference in age between the Black and MENA groups.

#### Gender

Approximately 59% of the entire sample were female. The White sample had the highest female make-up of 68%, followed by the Black sample which comprised 72% females, and the MENA sample which only comprised 36% females. The differences in gender makeup across ethnic groups were significant, *F*(2,597) = 35.46, *p* < 0.001, *R*^2^ = 0.1. Bonferroni analyses revealed that the MENA group’s gender makeup significantly differed from the Black and White groups, and that there were no significant differences in gender makeup between the Black and White groups.

#### Education

Participants averaged roughly 14 years (sd = 2.93) of education, meaning the average participant had at least some college education. The most educated group was the White sample, who averaged 14.8 years (sd = 2.11) of education, followed by the Black sample who averaged 14.6 years (sd = 2.02) of education, followed by the MENA group who averaged 13.4 years (sd = 4.02) of education. These differences in education were significant across ethnic groups, F(2,602) = 13.49, *p* < 0.001, *R*^2^ = 0.04. Post hoc Bonferroni analyses revealed that these ethnic differences in education significantly differed between the MENA and White groups and the MENA and Black groups. There was no significant difference in education between the Black and White group.

#### Income

We assessed income based on the binned scale mentioned in the methods section. Across the entire sample, participants reported a median income of 5. When looking at the ethnic differences in income, White and MENA participants reported a median income of 5, while Black participants reported a median income of 4. A Kruskal–Wallis analysis revealed that there were significant differences by ethnicity in median monthly income across ethnic groups, χ^2(2)^ = 11.21, *p* < 0.05. A post-hoc Wilcox test revealed that these significant differences were between Black and White participants, and between White and MENA participants.

#### Language

All participants who completed the interview in Arabic were MENA. Of these MENA participants, roughly 36% of participants completed the interview in Arabic.

### Research Question 1: Are there ethnic differences in physical and mental health status?

#### Mental Health

Out of all participants, 584 completed the CES-D-10. Of these participants, roughly 45 participants (7.5%) reported no depressive symptoms and 186 (31%) of participants met the threshold for depression, meaning they scored 10 or more on the measure. Across ethnic groups, 68 (35.8%) of all MENA participants, 62 (31%) of all Black participants, and 56 (28.9%) of all White participants did meet the threshold for depression. Path analyses revealed that there were no significant ethnic differences in mental health prevalence based on the raw, continuous scores of the CESD-10 across all ethnic groups.

#### Physical Health

Out of all participants, 598 reported their chronic health status. Of these participants, 142 (23.7%) reported having no chronic illness, 222 (37.1%) reported having one chronic illness, and 234 (39.1%) reported having two or more chronic illnesses. Across ethnic groups, 143 (73%) of all White participants, 170 (83.7%) of all Black participants, and 143 (71.9%) of all MENA participants had at least one chronic illness. Path analyses revealed that there were significant ethnic differences in chronic health status, as being Black was associated with having a higher number of chronic illnesses, when compared to White participants, *b* = 0.31, *p* < 0.01 and MENA participants, *b* = 0.42, *p* < 0.001.

### Research Question 2: Are there ethnic differences in cognitive performance?

#### Episodic Memory

On average, the White group showed the highest performance on the CERAD, averaging a z-score of 0.29 (sd = 1.76), followed by the Black group, who averaged a z-score of 0.17 (sd = 1.77). The MENA group had the lowest performance, as their average z-score was −0.34 (sd = 1.94). Path analyses revealed that Black participants performed worse when compared to their White counterparts, *b* = *−0.35, p* < *0.05*. There were no significant differences between MENA participants and other groups.

#### Working Memory

On the number span test, the White group showed the highest performance, averaging a score of 1.79 (sd = 0.46), followed by the Black group who averaged a score of 1.8 (sd = 0.46), followed by the MENA group, who averaged a score of 1.57 (sd = 0.66). Path analyses revealed that MENA participants scored significantly worse than White participants, *b* = *−0.17, p* < *0.01* and MENA participants scored significantly worse than Black participants, *b* = *−0.14, p* < *0.05*.

#### Verbal Fluency

For the verbal fluency test, the White group showed the highest performance, with an average score of 17.98 (sd = 6.59), followed by the Black group who had an average score of 15.8 (sd = 5.52), followed by the MENA group, who had an average score of 12.98 (sd = 6.57). Path analyses revealed that performance in both Black, *b* = *−2.6, p* < *0.001* and MENA, *b* = *−3.8, p* < *0.001* participants performed significantly worse than White participants.

### Research Question 3: Does ethnicity moderate the relationship between physical and mental health status and cognitive performance?

#### Mental Health (Table 2)

##### Episodic Memory (Fig. 1)

The chi-square fit statistic indicated that our full model, which included the interaction of ethnicity and depressive symptoms, where White participants were the reference group, was a better fit than the model that did not include the interaction, χ^2(2)^ = 9.6, *p* < 0.01. Within the model, the interaction between being Black and having depressive symptoms (*b* = 0.07) is significant, highlighting that there are differences in the relationship between depressive symptoms and episodic memory performance when comparing Black and White participants. To test MENA and Black differences, we set up contrasts where we compared coefficients of Black and MENA participants. When looking at the slopes of this interaction, we see that White participants have a significant slope of −0.075, *p* < 0.001 and MENA participants have a significant slope of −0.095, *p* < 0.001. Black participants had a non-significant slope of −0.006 (*p* > 0.05). These slopes indicate there is a stronger negative association between depressive symptoms and episodic memory performance for both White and MENA participants, but not for Black participants. Because the slope for Black participants is not significant, we cannot conclude that depressive symptoms are associated with episodic memory performance for Black participants.

##### Working Memory

The chi-square fit statistic indicated that our restricted model, which did not include interactions of ethnicity and depressive symptoms, was the best fit for our data, χ^2(2)^ = 1.7, *p* < 0.05. This indicates that the relationship between depressive symptoms and working memory performance did not differ by ethnicity.

##### Verbal Fluency

The chi-square fit statistic indicated that our restricted model, which did not include interactions of ethnicity and depressive symptoms, was a better fit than our full model, χ^2(2)^ = 4.3, *p* < 0.05. This indicates that the relationship between depressive symptoms and working memory performance did not differ by ethnicity.

##### Physical Health (Table 3)

###### Episodic Memory

The chi-square fit statistic indicated that our restricted model, which did not include interactions of ethnicity and chronic illness, was a better fit than our full model, χ^2(2)^ = 1.3, *p* < 0.05. There were no significant differences in slopes across ethnic and racial groups.

###### Working Memory

The chi-square fit statistic indicated that our restricted model, which did not include interactions of ethnicity and chronic illness, was a better fit than our full model, χ^2(2)^ = 1.7, *p* < 0.05. This indicates that the relationship between depressive symptoms and working memory performance did not differ by ethnicity.

###### Verbal Fluency

The chi-square fit statistic indicated that our restricted model, which did not include interactions of ethnicity and chronic illness, was a better fit than our full model, χ^2(2)^ = 0.4, *p* < 0.05. This indicates that the relationship between depressive symptoms and working memory performance did not differ by ethnicity.

## Discussion

The present study investigates ethnic differences in physical and mental health status, ethnic difference in cognitive health, and the extent to which ethnicity moderates the relationship between physical and mental health status and cognition among Black, White, and MENA older adults. Hypothesis 1 was partially supported, as we found that there were ethnic differences in chronic illness prevalence, where Black participants were more likely to have a chronic illness than their MENA and White counterparts. We did not find ethnic differences in depressive symptomatology. Next, Hypothesis 2 was fully supported, as we also found ethnic differences in all three domains of cognitive health, where White participants scored highest in all three domains of cognition. Finally, Hypothesis 3 was partially supported, as we did see racial and ethnic differences in our interactions, although not in the direction we expected. Although ethnicity moderated the relationship between depressive symptoms and episodic memory when comparing Black and White participants, the association was not stronger among Black participants, when compared to White participants, as hypothesized. Instead, the negative association between depressive symptoms and episodic memory was stronger among White participants when compared to Black participants. Additionally, the main moderation effect was not significant when looking at the interaction of race and depressive symptoms on working memory and verbal fluency, nor was there a significant main moderation effect when looking at the interaction of race and chronic illness on all three domains of cognition. It is important to note that these observed racial differences in health and cognition are likely a function of racial differences in socioeconomic status, due to the fact that socioeconomic status is an established mediator of race and health disparities [89].

These findings are partially consistent with existing literature and theoretical frameworks. Differences in chronic illness prevalence across groups are consistent with the Environmental Affordances Model, as systemic factors, such as inequality of income and education, put ethnic minority groups at a higher disposition for chronic illness. There is evidence that minority-related stressors, including implicit and explicit racism, increase the risk of developing a chronic illness at a higher magnitude for Black people when compared to their White counterparts [90]. However, these do not explain differences between Black and MENA participants, as MENA participants also face minority based stressors [91]. It is possible that these differences can be attributed to differences in the unique experiences faced by each group. For example, the aftermath of 9/11 has resulted in increased racial profiling while flying for MENA Americans [92], increased surveillance in predominantly Arab neighborhoods by institutions like the FBI, and an increase in hate crimes [93]. MENA instances of racial profiling differ from racial profiling on Black Americans in that they are more targeted and often at the Federal level. In contrast, Black Americans are more likely to face racial profiling while driving[94] [] and shopping [95] as a result of the lasting legacy of slavery. This type of profiling is likely to happen more often, and for some is seen as a daily occurrence[96]. Future research should explore these nuances in detail in order to give us a better understanding of minority-based health disparities.

Our findings, which showed no racial and ethnic differences in depressive symptoms, are consistent with Affordances Model, which posits that disadvantaged groups are more likely to engage in unhealthy coping behaviors, such as drinking and smoking, to cope with stress. This may be due to the stigmatization of depression in the Black community, as Black older adults are less likely to seek therapy and more likely to engage in coping behaviors due to fear of judgment from the community [97]. Similarly, depression is stigmatized in the MENA community, as MENA Americans are less likely to seek mental health treatment for depression and anxiety [98]. Both Black and MENA older adults also faced additional vulnerability during the pandemic, in addition to their existing minority based stressors. Specifically, Black Michiganders faced a higher burden of COVID-19 related deaths when compared to their White counterparts [99] and MENA older adults aged 80 and older residing in Michigan had a higher mortality rate when compared to their White and Black counterparts [100], which would increase the amount of worry and grief in these populations. However, this increase in stress likely increased the amount of coping behaviors in Black and MENA participants, which in turn eliminated disparities in depressive symptoms.

The observed differences in cognitive performance are consistent with our hypothesis and with the STAC-r framework. The literature highlights that both MENA and Black older adults are more likely to face adverse experiences over the lifespan, including socioeconomic disadvantage, unique stressors, and chronic illness[101–104] which are documented to negatively impact late life cognition [105, 106]. With respect to our study, the adverse effects of negative life course factors on cognition were seen in our sample, as the effects of chronic illness and depressive symptoms had more of a negative effect on the cognitive health of MENA older adults, who showed worse performance across each domain of cognition. These findings highlight unique risk factors of cognitive health for the MENA population, who are understudied in aging research. Further understanding and support in physical and mental health for the MENA population would be a first step of intervention as it pertains to improving cognitive health outcomes in the MENA community.

The moderating effect of ethnicity on the relationship between depressive symptoms and episodic memory, is somewhat consistent with both frameworks. On one hand, ethnic disparities in cognitive outcomes may be influenced by minority related stressors, which are more likely to be faced by ethnic minority groups over the course of the lifespan [107]. Institutional structures influence racism and discrimination, which adversely affects ethnic minority groups and their physical and mental health outcomes [108]. However, these findings do not take into account that the experiences of different minority groups differ. Although both MENA and Black people face racial and ethnic minority related stress, including prejudice and discrimination, there are also specific nuances in their unique experiences. For example, the minority related stress for the MENA population in the United States is also characterized by bicultural experience and acculturation as a result of post 9/11 discrimination [109], while the minority related stress of Black Americans is characterized by intergenerational disenfranchisement through systems and laws dating back to slavery [110]. Our findings emphasize the importance of studying differences between racial and ethnic minority groups because by not doing so, we miss important targets for intervention.

Our findings reflect both the temporal and geographical context of our study. Within the city of Detroit, where 80% of the population is Black, 89% of older adults have at least one chronic illness and 39% of older adults have 3 or more chronic illnesses [111]. These disparities are also reflected in pandemic related trends, as Black residents within the metro-Detroit region were at the highest risk of COVID-19 mortality due to comorbidities [112]. Similarly, MENA residents within metro-Detroit showed a higher mortality rate than their White counterparts [99]. These effects of the pandemic influenced health disparities in metro-Detroit during the pandemic, as there is evidence that COVID-19 related stress negatively impacted cognitive health of Black and MENA older adults living in metro-Detroit [60].The lack of disparities in depressive symptoms during the pandemic may be due to increased coping behaviors. A studied based in the Midwest showed that older adults coped during the pandemic by staying busy, relying on social networks, and maintaining a positive mindset [113].

These findings are also consistent with health disparities prior to the pandemic. Prior studies have highlighted that Black older adults show a higher chronic disease burden than other racial groups, including cardiovascular disorders [114, 115] and diabetes [116]. Further, MENA Americans self-reported worse cognitive health outcomes prior to the onset of the pandemic [117]. There is evidence that increased levels of stress serve as a risk factor for adverse physical and cognitive health status [118–120]. With this in mind, we can conclude that although the pandemic did not cause health disparities, it highlighted and potentially worsened pre-existing disparities.

Although these findings give valuable insight of cognitive outcomes in older adults in context of the pandemic, a limitation of our results is that they are cross sectional. As a result, we cannot address how cognitive outcomes may have developed through the course of the pandemic. Our sample is also more educated than the national population, as our sample averages 14 years of education, with roughly 75% of our sample reporting at least some college education in comparison to the national rate of 50% for this age group [121]. Given this, it would be important to understand cognitive outcomes in populations with fewer years of education which may be less resilient to factors that adversely affect cognition.

Follow-up work should extend this study further into the post pandemic period with the same pool of participants in order to characterize how cognitive abilities have developed as normalcy has resumed. Indeed, there are limitations to doing so, including attrition, but such a study will allow us to compare cognitive outcomes at different time points. This is important because it will allow us to understand how cognitive functioning among older adults has manifested over the course of the pandemic. This will allow us to understand the extent to which the pandemic has influenced cognitive aging trajectories.

Finally, further research is necessary to better understand the unique factors that place MENA older adults at elevated risk for poor cognitive health. This study suggests that chronic illness and depressive symptoms may be especially important risk factors in the cognitive health of this population. Future work should examine these risk factors further, alongside unique factors to the MENA experience, in order to identify meaningful targets for intervention. Doing so will be essential for addressing cognitive health disparities and ensuring that the needs of MENA communities are sufficiently represented in aging research.

## Figures and Tables

**Fig. 1:**
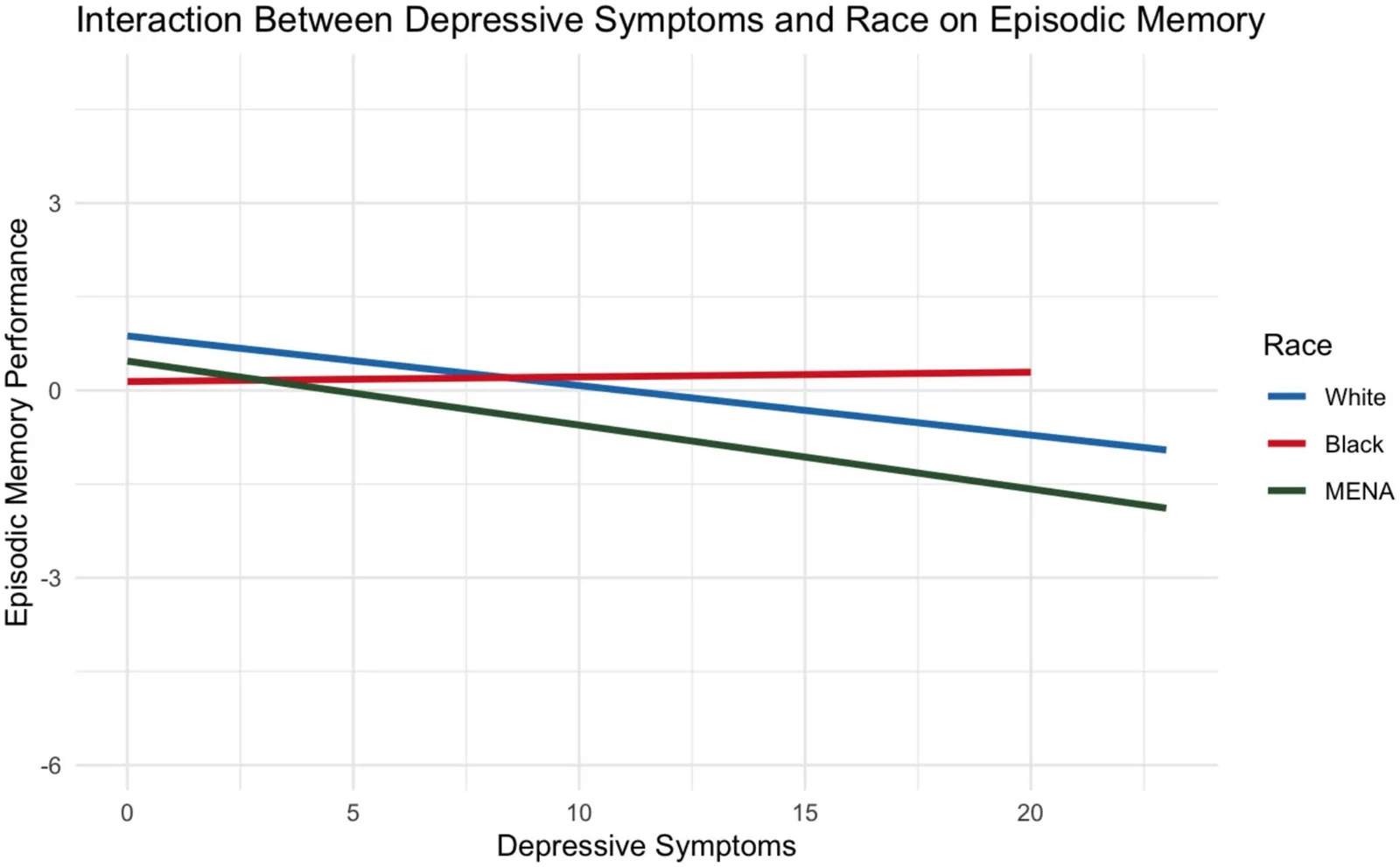
Fig. 1 depicts the interaction between race and depressive symptoms on episodic memory across groups. Among White participants (blue line), and MENA participants (green line), higher depressive symptoms are significantly associated with lower episodic memory performance. In contrast, the slope for Black participants (red line) is close to 0 and not statistically significant, suggesting that depressive symptoms are not significantly related to memory performance in this group. The raw number of depressive symptoms is represented on the x-axis and the Episodic Memory score for performance is represented on the y-axis. This figure was created in the ggplot package in RStudio

## Data Availability

Our data and analytic methods are available to other researchers for replication purposes upon request. Analyses reported in the manuscript were not pre registered.

## Code Availability

Our code is available to other researchers for replication purposes upon request. Analyses reported in the manuscript were not pre registered.

## Appendix

**Table 1:** Demographics

	Full Sample (*n* = 600)	MENA (*n* = 199)	Black (*n* = 205)	White (*n* = 196)	*P*-value
**Sociodemographic Variables**					
Age (64–94)	72.57 (6.58)	71.36 (6.07)	71.97 (5.95)	74.341(7.28)	0.001
Female N (%)	353 (59%)	72 (36%)	147 (72%)	134 (68%)	0.001
Education (sd)	14.25 (2.93)	13.4 (4.02)	14.59 (2.02)	14.76 (2.11)	0.001
Monthly Income (%) in range					0.05
> $500	1	3.25	0.63	0	
$501–1,000	6	9.74	8.86	1.99	
$1,001–2,000	12	21.43	14.56	11.92	
$2,001–3,000	16	14.94	27.22	21.85	
$3,001–5,000	18	18.83	24.69	27.81	
$5,001–10,000	18	20.78	20.25	28.47	
$10,000 <	6	11.04	3.80	7.95	
**Health Related Variables**					
Depressive Symptoms [0–23]	7.40 (5.20)	7.98 (5.30)	7.15 (5.19)	7.07 (5.09)	> 0.05
Chronic Illness [0–4]	1.29 (1.00)	1.19 (0.99)	1.46 (0.95)	1.21 (1.04)	0.01
**Cognitive Variables**					
Episodic Memory [−5.9—4.9]	0.04 (1.84)	−0.34 (1.94)	0.l7 (1.77)	0.29 (1.76)	0.05
Working Memory [0–2]	1.72 (0.54)	1.57 (0.66)	1.79 (0.46)	1.8 (0.46)	0.05
Verbal Fluency [0–35]	15.5 (6.58)	12.98 (6.57)	15.8 (5.52)	17.98 (6.59)	0.001

**Table 2:** Effects of mental health status on cognitive health

	Episodic Memory		Working Memory		Verbal Fluency	
Predictor	b	se	b	se	b	se
Intercept	1.255***	(0.2)	1.868***	(0.004)	19.35***	(0.586)
Depressive Symptoms	−0.075***	(0.023)	−0.01	(0.004)	−0.102*	(0.049)
Ethnicity (Black)	−0.834**	(0.284)	−0.027	(0.053)	−2.626***	(0.603)
Ethnicity (MENA)	−0.211	(0.306)	−0.170**	(0.063)	−3.769***	(0.710)
Gender (Male)	−0.909***	(0.147)	0.035	(0.046)	−1.079**	(0.527)
Age	−0.078***	(0.011)	−0.006	(0.003)	−0.176***	(0.039)
Language (Arabic)	0.382	(0.257)	−0.227**	(0.079)	−3.655***	(0.899)
Depressive Symptoms*Black	0.069*	(0.032)				
Depressive Symptoms*MENA	−0.02	(0.033)				
N	600		600		600	
Adjusted R^2^	0.33		0.07		0.17	
F-Statistic	128.76		45.53		114.64	

Significance codes: 0.001 ‘***’, 0.01 ‘**’, 0.05 ‘*’.

**Table 3:** Effects of physical health status on cognitive health

	Episodic Memory		Working Memory		Verbal Fluency	
Predictor	b	se	b	se	b	se
Intercept	0.857***	(0.152)	1.868***	(0.004)	18.91***	(0.539)
Chronic Illness	−0.137*	(0.071)	−0.011	(0.004)	−0.281	(0.252)
Ethnicity (Black)	−0.309	(0.284)	−0.032	(0.053)	−2.548***	(0.610)
Ethnicity (MENA)	−0.375	(0.306)	−0.172**	(0.063)	−3.828***	(0.713)
Gender (Male)	−0.813***	(0.147)	0.043	(0.046)	−0.891	(0.529)
Age	−0.078***	(0.011)	−0.007*	(0.003)	−0.171***	(0.039)
Language (Arabic)	0.661**	(0.257)	−0.26**	(0.079)	−3.927***	(0.889)
N	600		600		600	
Adjusted R^2^	0.17		0.06		0.17	
F-Statistic	128.76		40.47		108.13	

Significance codes: 0.001 ‘***’, 0.01 ‘**’, 0.05 ‘*’.

## References

[1] Smith M, Vaughan Sarrazin M, Wang X, Nordby P, Yu M, DeLonay AJ, Risk from delayed or missed care and non-COVID-19 outcomes for older patients with chronic conditions during the pandemic. J Am Geriatr Soc. 2022. 10.1111/jgs.17722. PMC9106879 35211958

[2] Briggs R, McDowell CP, De Looze C, Kenny RA, Ward M. Depressive symptoms among older adults pre–and post–COVID-19 pandemic. J Am Med Dir Assoc. 2021. 10.1016/j.jamda.2021.09.003. PMC8436876 34597531

[3] Chodosh J, Miller-Martinez D, Aneshensel CS, Wight RG, Karlamangla AS. Depressive symptoms, chronic diseases, and physical disabilities as predictors of cognitive functioning trajectories in older Americans. J Am Geriatr Soc. 2010. 10.1111/j.1532-5415.2010.03171.x. PMC3058867 21087219

[4] Reuter-Lorenz PA, Park DC. How does it STAC up? Revisiting the scaffolding theory of aging and cognition. Neuropsychol Rev. 2014. 10.1007/s11065-014-9270-9. PMC4150993 25143069

[5] Rose EJ, Ebmeier KP. Pattern of impaired working memory during major depression. J Affect Disord. 2006. 10.1016/j.jad.2005.11.003. 16364451

[6] Salazar-Villanea M, Liebmann E, Garnier-Villarreal M, Montenegro-Montenegro E, Johnson DK. Depressive symptoms affect working memory in healthy older adult hispanics. Journal of depression & anxiety. 2015. 10.4172/2167-1044.1000204. PMC4836854 27104091

[7] Evans J, Charness N, Dijkstra K, Fitzgibbons JM, Yoon J-S. Is episodic memory performance more vulnerable to depressive affect in older adulthood? Aging Neuropsychol Cogn. 2019(2). 10.1080/13825585.2018.1424314. PMC6192852 29310514

[8] Pauls F, Petermann F, Lepach AC. Episodic memory and executive functioning in currently depressed patients compared to healthy controls. Cogn Emot. 2015. 10.1080/02699931.2014.915208. 24828417

[9] Wasserman JS, Holtzer R. Depressive symptoms are associated with decline over time in verbal fluency performance in female but not male community-dwelling older adults. Exp Aging Res. 2024. 10.1080/0361073X.2023.2195295. PMC10539484 36989442

[10] Weisenbach SL, Boore LA, Kales HC. Depression and cognitive impairment in older adults. Curr Psychiatry Rep. 2012. 10.1007/s11920-012-0278-7. 22622478

[11] Shimada H, Park H, Makizako H, Doi T, Lee S, Suzuki T. Depressive symptoms and cognitive performance in older adults. J Psychiatr Res. 2014. 10.1016/j.jpsychires.2014.06.004. 25023083

[12] Goldstein FC, Levey AI, Steenland NK. High blood pressure and cognitive decline in mild cognitive impairment. J Am Geriatr Soc. 2013. 10.1111/jgs.12067. PMC3699694 23301925

[13] McCrimmon RJ, Ryan CM, Frier BM. Diabetes and cognitive dysfunction. Lancet. 2012. 10.1016/S0140-6736(12)60360-2. 22683129

[14] Obisesan TO, Obisesan OA, Martins S, Alamgir L, Bond V, Maxwell C, High blood pressure, hypertension, and high pulse pressure are associated with poorer cognitive function in persons aged 60 and older: the third national health and nutrition examination survey. J Am Geriatr Soc. 2008. 10.1111/j.1532-5415.2007.01592.x. PMC2614341 18179496

[15] Grober E, Hall CB, Hahn SR, Lipton RB. Memory impairment and executive dysfunction are associated with inadequately controlled diabetes in older adults. J Prim Care Community Health. 2011. 10.1177/2150131911409945 PMC3695412 23804840

[16] Sadanand S, Balachandar R, Bharath S. Memory and executive functions in persons with type 2 diabetes: a meta-analysis. Diabetes/metabolism research and reviews. 2016. 10.1002/dmrr.2664. 25963303

[17] Cansino S, Torres-Trejo F, Estrada-Manilla C, Mercado-Canales A, Medina-Velázquez D, Esquivel-García R, Ruiz-Velasco S. Effects of hypotension and hypertension on source memory and working memory. Aging Ment Health. 2022. 10.1080/13607863.2021.1942435. 34225518

[18] Fujiwara T, Hoshide S, Kanegae H, Eguchi K, Kario K. Exaggerated blood pressure variability is associated with memory impairment in very elderly patients. J Clin Hypertens. 2018. 10.1111/jch.13231. PMC8031126 29466618

[19] Saxby BK, Harrington F, McKeith IG, Wesnes K, Ford GA. Effects of hypertension on attention, memory, and executive function in older adults. Health Psychol. 2023. 10.1037/0278-6133.22.6.587. 14640855

[20] Godefroy O, Roussel M, Leclerc X, Leys D. Deficit of episodic memory: anatomy and related patterns in stroke patients. Eur Neurol. 2009. 10.1159/000197107. 19176963

[21] Lugtmeijer S, Lammers NA, de Haan EH, de Leeuw FE, Kessels RP. Post-stroke working memory dysfunction: a meta-analysis and systematic review. Neuro Psychol Rev. 2021. 10.1007/s11065-020-09462-4. PMC7889582 33230717

[22] Spaccavento S, Marinelli CV, Nardulli R, Macchitella L, Bivona U, Piccardi L, Attention deficits in stroke patients: the role of lesion characteristics, time from stroke, and concomitant neuropsychological deficits. Behav Neurol. 2019. 10.1155/2019/7835710. PMC6556322 31263512

[23] Hochstenbach J, Mulder T, Van Limbeek J, Donders R, Schoonderwaldt H. Cognitive decline following stroke: a comprehensive study of cognitive decline following stroke*. J Clin Exp Neuropsychol. 1998. 10.1076/jcen.20.4.503.1471. 9892054

[24] Mezuk B, Abdou CM, Hudson D, Kershaw KN, Rafferty JA, Lee H, “White Box” epidemiology and the social neuroscience of health behaviors: The environmental affordances model. Soc Ment Health. 2013. 10.1177/215686931348089. PMC3820104 24224131

[25] Hooker K, Phibbs S, Irvin VL, Mendez-Luck CA, Doan LN, Li T, Depression among older adults in the United States by disaggregated race and ethnicity. Gerontologist. 2019. 10.1093/geront/gny159. PMC7729206 30561600

[26] Abuelezam NN, El-Sayed AM, Galea S. The health of Arab Americans in the United States: an updated comprehensive literature review. Front Public Health. 2018. 10.3389/fpubh.2018.00262. PMC6141804 30255009

[27] Jamil H, Grzybowski M, Hakim-Larson J, Fakhouri M, Sahutoglu J, Khoury R, Factors associated with self-reported depression in Arab, Chaldean, and African Americans. Ethn Dis. 2008;18(4):464–70. 19157251

[28] Chatters LM, Taylor RJ, Woodward AT, Nicklett EJ. Social support from church and family members and depressive symptoms among older African Americans. Am J Geriatr Psychiatry. 2015. 10.1016/j.jagp.2014.04.008. PMC4216772 24862679

[29] Benca-Bachman CE, Najera DD, Whitfield KE, Taylor JL, Thorpe RJ Jr, Palmer RH. Quality and quantity of social support show differential associations with stress and depression in African Americans. Am J Geriatr Psychiatry. 2020. 10.1016/j.jagp.2020.02.004. PMC7246182 32165073

[30] Dallo FJ, Prabhakar D, Ruterbusch J, Schwartz K, Peterson EL, Liu B, Screening and follow-up for depression among Arab Americans. Depress Anxiety. 2018. 10.1002/da.22817. 30099819

[31] Quiñones AR, Botoseneanu A, Markwardt S, Nagel CL, Newsom JT, Dorr DA, Racial/ethnic differences in multimorbidity development and chronic disease accumulation for middle-aged adults. PLoS ONE. 2019. 10.1371/journal.pone.0218462. PMC6576751 31206556

[32] Office of Minority Health. Heart Disease and African Americans. 2022. https://minorityhealth.hhs.gov/heart-disease-and-african-americans#:~:text=In%202019%2C%20African%20Americans%20were Accessed 13 December 2024

[33] Davis J, Penha J, Mbowe O, Taira DA. Peer reviewed: prevalence of single and multiple leading causes of death by race/ethnicity among US adults aged 60 to 79 years. Preventing Chronic Disease. 2017. 10.5888/pcd14.160241. PMC5652239 29049018

[34] Ostchega Y, Fryar CD, Nwankwo T, Nguyen DT. (2020). Hypertension prevalence among adults aged 18 and over: United States, 2017–2018. In: NCHS Data Brief. 2020. https://www.cdc.gov/nchs/data/databriefs/db364-h.pdf. Accessed 13 December 2024 32487290

[35] Abuelezam NN, El-Sayed AM, Galea S. Differences in health behaviors and health outcomes among non-Hispanic Whites and Arab Americans in a population-based survey in California. BMC Public Health. 2019. 10.1186/s12889-019-7233-z. PMC6613261 31286920

[36] Fritz H, DiZazzo-Miller R, Bertran EA, Pociask FD, Tarakji S, Arnetz J, Diabetes self-management among Arab Americans: patient and provider perspectives. BMC Int Health Hum Rights. 2016. 10.1186/s12914-016-0097-8. PMC5006513 27582174

[37] Bertran EA, Fritz H, Abbas M, Tarakji S, DiZazzo-Miller R, Pociask FD, The impact of Arab American culture on diabetes self-management education. Diabetes Educ. 2015. 10.1177/0145721715607356. 26450219

[38] Schulz AJ, Mentz GB, Sampson N, Race and the distribution of social and physical environmental risk: a case example from the Detroit Metropolitan Area. Du Bois Rev Soc Sci Res Race. 2016. 10.1017/S1742058X16000163. PMC5610908 28951763

[39] Sugrue TJ. The origins of the urban crisis: Race and inequality in postwar detroit-updated edition. Princeton University Press; 2014;

[40] McClure E, Feinstein L, Cordoba E, Douglas C, Emch M, Robinson W, The legacy of redlining in the effect of foreclosures on Detroit residents’ self-rated health. Health Place. 2019. 10.1016/j.healthplace.2018.10.004. PMC6345551 30448354

[41] Dean CA, Milan R, Raja M, Radhakrishnan S. Reducing Detroit’s Heart Disease Risk: The Role of Social Determinants of Health and Health Behaviors. Michigan Journal of Public Health. 2024

[42] Heart disease exposes disparities, so medicine goes mobile in Detroit. 2022. Accessed 22 July 2025 https://bridgemi.com/michigan-health-watch/heart-disease-exposes-disparities-so-medicine-goes-mobile-detroit/

[43] Schulz AJ, Gravlee CC, Williams DR, Israel BA, Mentz G, Rowe Z. Discrimination, symptoms of depression, and self-rated health among African American women in Detroit: results from a longitudinal analysis. Am J Public Health. 2006. 10.2105/AJPH.2005.064543. PMC1483853 16735638

[44] Sol K, Morris EP, Lee JH, Zaheed AB, Palms JD, Scambray K, Histories of neighborhood socioeconomic status contribute to race differences in later-life cognition. Alzheimers Dement. 2024. 10.1002/alz.13786. PMC11095476 38552138

[45] Zahodne LB, Sol K, Scambray K, Lee JH, Palms JD, Morris EP, Neighborhood racial income inequality and cognitive health. Alzheimers Dement. 2024. 10.1002/alz.13911. PMC11350017 38934219

[46] American Community Survey 2015–2019 5-Year Data Release. 2020. Accessed 22 July 2025 https://www.census.gov/newsroom/press-kits/2020/acs-5-year.html

[47] Hekman K, Weir S, Fussman C, Lyon-Callo S. 2015. Health Risk Behaviors Among Arab Adults Within the State of Michigan: 2013 Arab Behavioral Risk Factor Survey. Lansing, MI: Michigan Department of Health and Human Services, Lifecourse Epidemiology and Genomics Division and Health Disparities Reduction and Minority Health Section.

[48] Neumayer H, Weir S, Fussman C, McKane P. 2017. Health Risk Behaviors Among Arab Adults Within the State of Michigan: 2016 Arab Behavioral Risk Factor Survey. Lansing, MI: Michigan Department of Health and Human Services, Lifecourse Epidemiology and Genomics Division and Health Disparities Reduction and Minority Health Section.

[49] Patel MR, Green M, Tariq M, A snapshot of social risk factors and associations with health outcomes in a community sample of Middle Eastern and North African (MENA) people in the U.S. J Immigr Minor Health. 2022;24:376–84. 10.1007/s10903-021-01176-w. PMC7948165 33704656

[50] Schwartz KL, Kulwicki A, Weiss LK, Fakhouri H, Sakr W, Kau G, Cancer among Arab Americans in the Metropolitan Detroit Area. Ethn Dis. 2004;14(1):141–6. 15002934

[51] El-Sayed AM, Galea S. The health of Arab-Americans living in the United States: a systematic review of the literature. BMC Public Health. 2009. 10.1186/1471-2458-9-272. PMC2728720 19643005

[52] Silva C, Fonseca C, Ferreira R, Weidner A, Morgado B, Lopes MJ, Depression in older adults during the COVID-19 pandemic: a systematic review. J Am Geriatr Soc. 2023;71(7):2308–25. 10.1111/jgs.18363 37029710

[53] Purtle J. COVID-19 and mental health equity in the United States. Soc Psychiatry Psychiatr Epidemiol. 2020;55:969–71. 10.1007/s00127-020-01896-8. PMC7298157 32556376

[54] Pyne JD, Brickman AM. The impact of the COVID-19 pandemic on dementia risk: potential pathways to cognitive decline. Neurodegener Dis. 2021;21(1–2):1–23. 10.1159/000518581. PMC8678181 34348321

[55] Greenaway C, Hargreaves S, Barkati S, Coyle CM, Gobbi F, Veizis A, COVID-19: exposing and addressing health disparities among ethnic minorities and migrants. J Travel Med. 2020. 10.1093/jtm/taaa113. PMC7454797 32706375

[56] Benitez J, Courtemanche C, Yelowitz A. Racial and ethnic disparities in COVID-19: evidence from six large cities. J Econ Race Policy. 2020. 10.1007/s41996-020-00068-9. PMC7584480 35300199

[57] Examining and Addressing COVID-19 Racial Disparities in Detroit. 2021. Accessed 22 July 2025 https://detroitsurvey.umich.edu/wp-content/uploads/2021/04/Detroit_Covid_report_final.pdf

[58] Sidahmed E, Homayouni R, Childers K, Lick D, Oleszkowicz A, Weitz E, Mulhem E. Disparities in SARS-CoV-2 Infection Among Arab Americans Living in Southeast Michigan. Journal of Racial and Ethnic Health Disparities. 2025; https:// 10.1007/s40615-024-02206-7 39994153

[59] Dallo FJ, Kindratt TB, Seaton R, Ruterbusch JJ. The disproportionate burden of COVID-19 cases among Arab Americans. J Racial Ethn Health Disparities. 2023. 10.1007/s40615-022-01298-3. PMC8992413 35394622

[60] Ajrouch KJ, Zahodne LB, Brauer S, Tarraf W, Antonucci TC. COVID-19 stress and cognitive disparities in Black, MENA, and White older adults. Gerontologist. 2024. 10.1093/geront/gnae073. PMC11266980 38853657

[61] Wright CB, DeRosa JT, Moon MP, Strobino K, DeCarli C, Cheung YK, Race/ethnic disparities in mild cognitive impairment and dementia: the Northern Manhattan Study. J Alzheimers Dis. 2021. 10.3233/JAD-201370. PMC8150441 33646162

[62] Kindratt TB, Dallo FJ, Zahodne LB, Ajrouch KJ. Cognitive limitations among Middle Eastern and North African immigrants. J Aging Health. 2022. 10.1177/08982643221103712. PMC9633450 35606926

[63] Sloan FA, Wang J. Disparities among older adults in measures of cognitive function by race or ethnicity. J Gerontol B Psychol Sci Soc Sci. 2005. 10.1093/geronb/60.5.P242. 16131618

[64] Zsembik BA, Peek MK. Race differences in cognitive functioning among older adults. J Gerontol B Psychol Sci Soc Sci. 2001. 10.1093/geronb/56.5.S266. 11522808

[65] Fyffe DC, Mukherjee S, Barnes LL, Manly JJ, Bennett DA, Crane PK. Explaining differences in episodic memory performance among older African Americans and Whites: the roles of factors related to cognitive reserve and test bias. J Int Neuropsychol Soc. 2011. 10.1017/S1355617711000476. PMC3496282 23131601

[66] Sutin AR, Stephan Y, Terracciano A. Verbal fluency and risk of dementia. Int J Geriatr Psychiatry. 2019. 10.1002/gps.5081. PMC6530594 30729575

[67] Zuelsdorff M, Okonkwo OC, Norton D, Barnes LL, Graham KL, Clark LR, Stressful life events and racial disparities in cognition among middle-aged and older adults. J Alzheimers Dis. 2020. 10.3233/JAD-190439. PMC7481054 31815690

[68] Ajrouch KJ, Tarraf W, Brauer S, Zahodne LB, Antonucci TC. Adapted MoCA for use among Arabic-speaking immigrants in the United States. J Cross-Cult Gerontol. 2024. 10.1007/s10823-024-09513-w. PMC11584311 39083173

[69] Zahodne LB, Brauer S, Tarraf W, Morris EP, Antonucci TC, Ajrouch KJ. Measurement and structural invariance of a neuropsychological battery among Middle Eastern/North African, Black, and White older adults. Neuropsychology. 2023. 10.1037/neu0000902. PMC10544699 36996172

[70] Stinson JM, Armendariz V, Hegazy MIR, Strutt AM, McCauley SR, York MK. Developing a culturally competent neuropsychological battery for diagnosis of dementia in Arabic-speaking patients in the United States. Arch Clin Neuropsychol. 2023. 10.1093/arclin/acad017. 36988467

[71] Kindratt TB, Smith A. Cognitive difficulty in Middle Eastern and North African adults living in the United States compared with other racial and ethnic categories, 2017–2021. Am J Public Health. 2024. 10.2105/AJPH.2024.307803. PMC11447784 39357001

[72] Al-Rousan T, Kamalyan L, Bernstein Sideman A, Miller B, AlHeresh R, Moore A, Migration and cognitive health disparities: the Arab American and refugee case. The Journals of Gerontology: Series B. 2023. 10.1093/geronb/gbac129. PMC9890904 36056890

[73] Kindratt TB, Ajrouch KJ, Zahodne LB, Dallo FJ. Suspected undiagnosed ADRD among Middle Eastern and North African Americans. J Immigr Minor Health. 2023. 10.1007/s10903-023-01509-x. PMC10527952 37351736

[74] Díaz-Venegas C, Downer B, Langa KM, Wong R. Racial and ethnic differences in cognitive function among older adults in the USA. Int J Geriatr Psychiatry. 2016. 10.1002/gps.4410. PMC4945484 26766788

[75] Zahodne LB, Sharifian N, Kraal AZ, Zaheed AB, Sol K, Morris EP, Socioeconomic and psychosocial mechanisms underlying racial/ethnic disparities in cognition among older adults. Neuropsychology. 2021. 10.1037/neu0000720. PMC8363204 33970660

[76] Vásquez E, Botoseneanu A, Bennett JM, Shaw BA. Racial/ethnic differences in trajectories of cognitive function in older adults: role of education, smoking, and physical activity. J Aging Health. 2016. 10.1177/0898264315620589. 26719488

[77] Socioeconomic Status. In: American Psychological Association. 2025. https://www.apa.org/topics/socioeconomic-status. Accessed 21 May 2025

[78] Shrider E. Poverty rate for the black population fell below pre-pandemic levels. In: Census.gov. 2023. https://www.census.gov/library/stories/2023/09/black-poverty-rate.html. Accessed 13 December, 2024

[79] Morgan I, Amerikaner A. Funding Gaps 2018: An Analysis of School Funding Equity across the U.S. and within Each State. In ERIC. Education Trust, 2018; https://eric.ed.gov/?id=ED587198

[80] McMaughan DJ, Oloruntoba O, Smith ML. Socioeconomic status and access to healthcare: interrelated drivers for healthy aging. Front Public Health. 2020. 10.3389/fpubh.2020.00231. PMC7314918 32626678

[81] Ohlson M. Effects of socioeconomic status and race on access to healthcare in the United States. Perspectives. 2020;12(1):2.

[82] Williams DR, John DA, Oyserman D, Sonnega J, Mohammed SA, Jackson JS. Research on discrimination and health: an exploratory study of unresolved conceptual and measurement issues. Am J Public Health. 2012. 10.2105/AJPH.2012.300702. PMC3329155 22420798

[83] Ward JB, Gartner DR, Keyes KM, Fliss MD, McClure ES, Robinson WR. How do we assess a racial disparity in health? Distribution, interaction, and interpretation in epidemiological studies. Ann Epidemiol. 2019. 10.1016/j.annepidem.2018.09.007. PMC6628690 30342887

[84] Antonucci TC, Akiyama H. Convoys of attachment and social relations in children, adolescents, and adults. Social networks and social support in childhood and adolescence. 1994. 10.1515/9783110866377.37.

[85] Heckathorn DD. Respondent-driven sampling: a new approach to the study of hidden populations. Soc Probl. 1997. 10.2307/3096941.

[86] Centers for Disease Control and Prevention. CDC museum COVID-19 timeline. https://www.cdc.gov/museum/timeline/covid19.html Accessed 13 Dec 2024

[87] Zhang W, O’Brien N, Forrest JI, Salters KA, Patterson TL, Montaner JSG, Validating a shortened depression scale (10 item CES-D) among HIV-positive people in British Columbia, Canada. PLoS ONE. 2012. 10.1371/journal.pone.0040793. PMC3400644 22829885

[88] VanderWeele TJ, Robinson WR. On the causal interpretation of race in regressions adjusting for confounding and mediating variables: Epidemiology. 2014. 10.1097/EDE.0000000000000105. PMC4125322 24887159

[89] Jackson JW. Explaining intersectionality through description, counterfactual thinking, and mediation analysis. Soc Psychiatry Psychiatr Epidemiol. 2017. 10.1007/s00127-017-1390-0. 28540515

[90] Splan ED, Magerman AB, Forbes CE. Associations of regional racial attitudes with chronic illness in the United States. Soc Sci Med. 2021. 10.1016/j.socscimed.2021.114077. 34126292

[91] Awad GH, Ikizler AS, Maghsoodi AH. Stress and Health Among Arab/MENA Americans. In Nassar SC, Ajrouch KJ, Dallo FJ, Hakim-Larson J, editors. Biopsychosocial Perspectives on Arab Americans. Springer International Publishing; 2023. pp 301–315. 10.1007/978-3-031-28360-4_16

[92] Considine C. The Racialization of Islam in the United States: Islam-ophobia, Hate Crimes, and “Flying while Brown.” Religions. 2017. 10.3390/rel8090165.

[93] Sun IY, Wu Y, Poteyeva M. Arab Americans’ opinion on counterterrorism measures: the impact of race, ethnicity, and religion. Stud Conflict Terror. 2011. 10.1080/1057610X.2011.578550.

[94] Bell GC, Hopson MC, Craig R, Robinson NW. Exploring black and white accounts of 21st-century racial profiling: riding and driving while black. Qual Res Rep Commun. 2014. 10.1080/17459435.2014.955590.

[95] Pittman C. “Shopping while Black”: Black consumers’ management of racial stigma and racial profiling in retail settings. J Consum Cult. 2020. 10.1177/1469540517717777.

[96] Banks KH, Kohn-Wood LP, Spencer M. An examination of the African American experience of everyday discrimination and symptoms of psychological distress. Community Ment Health J. 2006. 10.1007/s10597-006-9052-9. 16897412

[97] Conner KO, Copeland VC, Grote NK, Rosen D, Albert S, McMurray ML, Barriers to treatment and culturally endorsed coping strategies among depressed African-American older adults. Aging Ment Health. 2010. 10.1080/13607863.2010.501061. PMC3060025 21069603

[98] Jaber RM, Farroukh M, Ismail M, Najda J, Sobh H, Hammad A, Measuring depression and stigma towards depression and mental health treatment among adolescents in an Arab-American community. Int J Cult Ment Health. 2015. 10.1080/17542863.2014.953188. PMC4527177 26257824

[99] Parpia AS, Martinez I, El-Sayed AM, Wells CR, Myers L, Duncan J, Racial disparities in COVID-19 mortality across Michigan, United States. EClinicalMedicine. 2021. 10.1016/j.eclinm.2021.100761. PMC7933264 33718849

[100] Dallo FJ, Kindratt TB, Seaton R, Ruterbusch JJ. COVID-19 case-fatality rates in Michigan are higher for Arab Americans compared with non-Hispanic White individuals for the oldest age groups. Ethn Dis. 2025. 10.18865/EthnDis-2023-82. PMC11928025 40124640

[101] Abuelezam NN, El-Sayed AM, Galea S, Gordon NP. Health risks and chronic health conditions among Arab American and White adults in northern California. Ethn Dis. 2021. 10.18865/ed.31.2.235. PMC8054874 33883864

[102] Pampati S, Alattar Z, Cordoba E, Tariq M, Mendes De Leon C. Mental health outcomes among Arab refugees, immigrants, and U.S. born Arab Americans in Southeast Michigan: A cross-sectional study. BMC Psychiatry, 2018; 10.1186/s12888-018-1948-8 PMC6280467 30514261

[103] Ellis KR, Hecht HK, Young TL, Oh S, Thomas S, Hoggard LS, Chronic disease among African American families: a systematic scoping review. Prev Chronic Dis. 2020. 10.5888/pcd17.190431. PMC7784550 33416471

[104] Hudson DL, Neighbors HW, Geronimus AT, Jackson JS. Racial discrimination, John Henryism, and depression among African Americans. J Black Psychol. 2016. 10.1177/0095798414567757. PMC4903152 27529626

[105] Franks KH, Rowsthorn E, Bransby L, Lim YY, Chong TT-J, Pase MP. Association of self-reported psychological stress with cognitive decline: a systematic review. Neuropsychol Rev. 2023. 10.1007/s11065-022-09567-y. 36456767

[106] Ihle A, Ghisletta P, Ballhausen N, Fagot D, Vallet F, Baeriswyl M, Sauter J, Oris M, Maurer J, Kliegel M. The role of cognitive reserve accumulated in midlife for the relation between chronic diseases and cognitive decline in old age: a longitudinal follow-up across six years. Neuropsychologia. 2018. 10.1016/j.neuropsychologia.2018.10.013. 30359653

[107] Glymour MM, Manly JJ. Lifecourse social conditions and racial and ethnic patterns of cognitive aging. Neuropsychology Review. 2008. 10.1007/s11065-008-9064-z. 18815889

[108] Williams DR, Lawrence JA, Davis BA. Racism and health: evidence and needed research. Annu Rev Public Health. 2019;40(1):105. 10.1146/annurev-publhealth-040218-043750. PMC6532402 30601726

[109] Cainkar LA. Homeland Insecurity: the Arab American and Muslim American experience after 9/11: The Arab American and Muslim American Experience After 9/11. Russell Sage Foundation; 2011

[110] Cottrell D, Herron MC, Rodriguez JM, Smith DA. Mortality, incarceration, and African American disenfranchisement in the contemporary United States. Am Polit Res. 2019. 10.1177/1532673X18754555.

[111] BridgeDetroit publishes Dr. Smitherman op-ed on ‘Dying Before Their Time’ study. 2020. Accessed 22 July 2025; https://today.wayne.edu/medicine/news/2020/08/14/bridgedetroit-publishes-dr-smitherman-op-ed-on-dying-before-their-time-study-38857

[112] Parpia AS, Martinez I, El-Sayed AM, Wells CR, Myers L, Duncan J, Racial disparities in COVID-19 mortality across Michigan, United States. EClinicalMedicine. 2021. 10.1016/j.eclinm.2021.100761. PMC7933264 33718849

[113] Fuller HR, Huseth-Zosel A. Lessons in resilience: initial coping among older adults during the COVID-19 pandemic. Gerontologist. 2021. 10.1093/geront/gnaa170. PMC7665461 33136144

[114] Update AS. Heart disease and stroke statistics–2017 update. Circulation. 2017. 10.1161/CIR.0000000000000350.

[115] Movahed MR, John J, Hashemzadeh M, Jamal MM, Hashemzadeh M. Trends in the age adjusted mortality from acute ST segment elevation myocardial infarction in the United States (1988–2004) based on race, gender, infarct location and comorbidities. Am J Cardiol. 2009. 10.1016/j.amjcard.2009.05.051. 19801019

[116] National Diabetes Statistics Report, 2024. Accessed 22 July 2025; https://www.cdc.gov/diabetes/php/data-research/index.html

[117] Dallo FJ, Kindratt TB, Zahodne L. Prevalence of self-reported cognitive impairment among Arab American immigrants in the United States. Innov Aging. 2021. 10.1093/geroni/igaa058. PMC7788314 33442566

[118] Marin MF, Lord C, Andrews J, Juster RP, Sindi S, Arsenault-Lapierre G, Chronic stress, cognitive functioning and mental health. Neurobiol Learn Mem. 2011. 10.1016/j.nlm.2011.02.016. 21376129

[119] Lupien SJ, Maheu F, Tu M, Fiocco A, Schramek TE. The effects of stress and stress hormones on human cognition: implications for the field of brain and cognition. Brain Cogn. 2007. 10.1016/j.bandc.2007.02.007. 17466428

[120] Morris T, Moore M, Morris F. Stress and chronic illness: the case of diabetes. J Adult Dev. 2011. 10.1007/s10804-010-9118-3.

[121] Ryan CL, Bauman K. Educational Attainment in the United States: 2015 Population Characteristics Current Population Reports. US Census. 2016; https://www.census.gov/content/dam/Census/library/publications/2016/demo/p20-578.pdf

